# Integrating Genes Affecting Coronary Artery Disease in Functional Networks by Multi-OMICs Approach

**DOI:** 10.3389/fcvm.2018.00089

**Published:** 2018-07-17

**Authors:** Baiba Vilne, Heribert Schunkert

**Affiliations:** ^1^Deutsches Herzzentrum München, Klinik für Herz- und Kreislauferkrankungen, Technische Universität München, Munich, Germany; ^2^Munich Heart Alliance, German Centre for Cardiovascular Research, Munich, Germany

**Keywords:** cardiovascular disease, multiomics, genomics, transcriptomics, metabolomics, gut microbiome

## Abstract

Coronary artery disease (CAD) and myocardial infarction (MI) remain among the leading causes of mortality worldwide, urgently demanding a better understanding of disease etiology, and more efficient therapeutic strategies. Genetic predisposition as well as the environment and lifestyle are thought to contribute to disease risk. It is likely that non-linear and complex interactions occur between these multiple factors, involving simultaneous pathological changes in diverse cell types, tissues, and organs, at multiple molecular levels. Recent technological advances have exponentially expanded the breadth of available -omics data, from genome, epigenome, transcriptome, proteome, metabolome to even the microbiome. Integration of multiple layers of information across several -omics domains, i.e., the so-called multi-omics approach, currently holds the promise as a path toward precision medicine. Indeed, a more meaningful interpretation of genotype-phenotype relationships and the development of successful therapeutics tailored to individual patients are urgently needed. In this review, we will summarize recent findings and applications of integrative multi-omics in elucidating the etiology of CAD/MI; with a special focus on established disease susceptibility loci sequentially identified in genome-wide association studies (GWAS) over the last 10 years. Moreover, in addition to the autosomal genome, we will also consider the genetic variation in our “second genome”—the mitochondrial genome. Finally, we will summarize the current challenges in the field and point to future research directions required in order to successfully and effectively apply these approaches for precision medicine.

## Introduction

In the current era of high-potency statin therapy it becomes increasingly clear that even individuals with normal LDL-cholesterol levels without any conventional risk factors may develop atherosclerosis (1). The most pertinent manifestation of atherosclerosis is coronary artery disease (CAD), a highly complex disease, influenced by both multiple genetic risk variants and lifetime exposure to an atherogenic environment (2). A better understanding of the etiology of CAD and directions toward hitherto therapeutically not addressed disease mechanisms are urgently demanded (3). During the last 10 years, the genetic risk has been thoroughly explored in numerous genome-wide association studies (GWAS), leading to identification of >300 chromosomal loci which all significantly affect the risk of CAD (4–15). More than 90% of these common disease risk variants are located outside the protein-coding regions and have modest effect sizes (2, 16). Collectively they explain only ~25% of the overall disease heritability. This suggests that genetic variation may contribute to disease risk in a non-linear, interactive and complex way (17), leading to pathological changes in diverse cell types, tissues, and organs, at multiple molecular levels (18).

**Figure 1:**
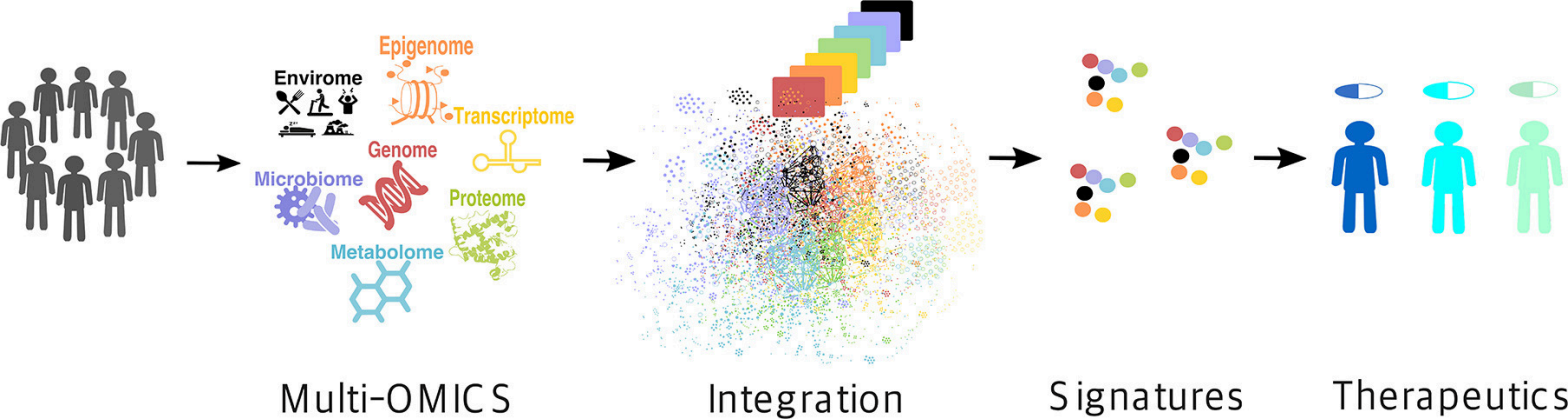
Multi-omics approach for precision medicine. Multi-omics (i.e., genome, epigenome, transcriptome, proteome, metabolome, microbiome, and envirome) data are collected from patients and integrated to create their individual molecular signatures (i.e., complex biomarkers), which are then used to select an appropriate drug for a particular patient, thus improving the treatment efficiency and reducing the possible side effects.

Recent technological advances have exponentially expanded the breadth of available -omics data (17). High-throughput monitoring of the abundance of various biological molecules and determination of their variation between different conditions on a global scale has become possible, promoting a paradigm shift in the way we approach biomedical problems (19). At the same time, it has been increasingly recognized that no single type of data can fully capture the intricacy of most complex molecular traits that manifest collectively as disease phenotypes (20–22). Rather, it is the integration of multiple layers of information across several -omics domains, i.e., the so-called multi-omics approach [also referred to as integromics or panomics (19)], that holds the promise for precision medicine (Figure 1) (19).

**Figure 2:**
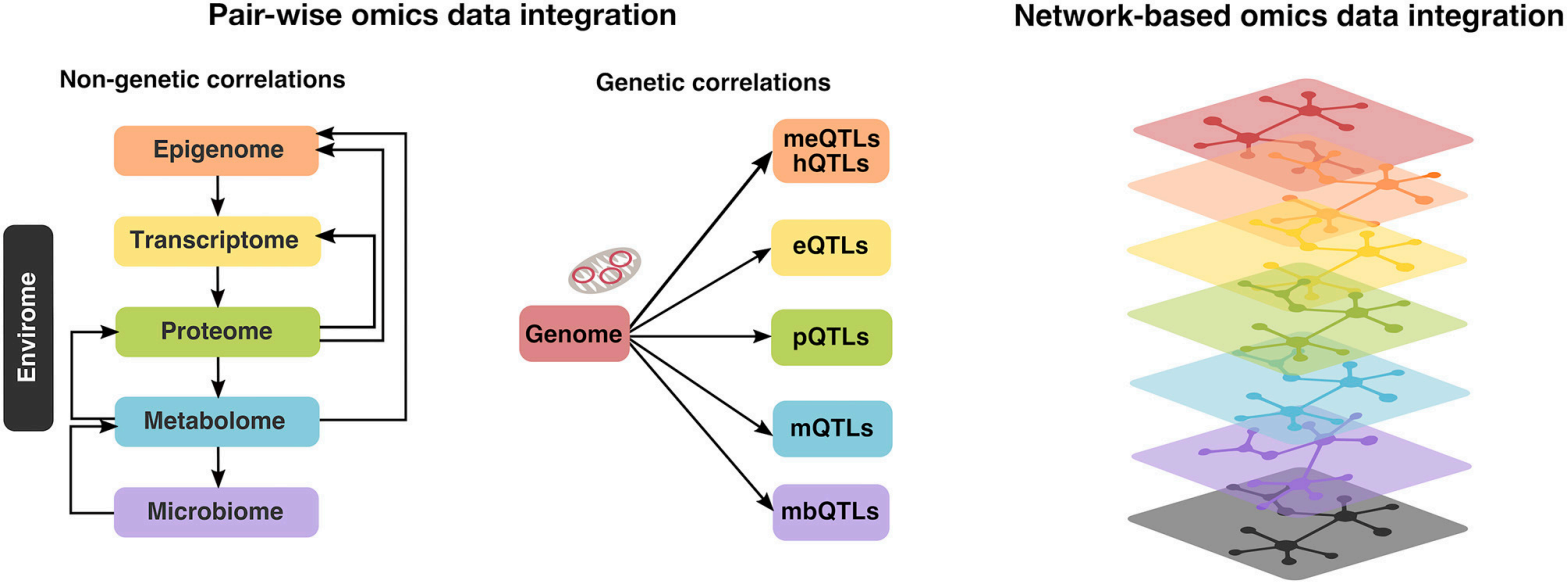
Multi-omics (i.e., autosomal and mitochondrial genome, epigenome, transcriptome, proteome, metabolome, microbiome, and envirome) data integration can be conducted in two ways: pair-wise integrations, which can be further divided into non-genetic (left panel) and genetic correlations (middle panel). In the first case, one would examine the correlation patterns between the down-stream omics layers (e.g., metabolome and gut microbiome), whereas the second is achieved via the so called quantitative trait loci (QTL) mapping, linking genetic variation to methylation levels (meQTLs) or histone modifications (hQTLs), transcriptome (expression QTLs; eQTLs), protein (pQTLs), metabolite (mQTLs) or measures of microbiome (mbQTLs). Alternatively, multi-dimensional i.e., network-based integration approaches (right panel) exist, however their application in the context of CAD has so far been limited (22).

Of note, integrative analysis across multiple-omics layers can be conducted in two ways (Figure 2): pair-wise data integration and multi-dimensional i.e., network-based integration (22). Furthermore, pair-wise integrations can be divided into genetic and non-genetic correlations (22). In the first case, DNA variants (i.e., allelic distributions of single-nucleotide polymorphisms; SNPs) are tested for association with down-stream omics markers such as transcriptomic alterations, protein, metabolite or methylation levels or quantitative and qualitative measures of microbiome, via the so called quantitative trait loci (QTL) mapping. In the second scenario, one would explore correlations between down-stream omics data, e.g., correlation of CpG methylation levels to transcript expression or between metabolome and gut microbiome, however it may be difficult to infer causal relationships in such case (22). Considering the largely unexplored role of the established CAD risk loci from GWAS (23) and the central dogma that genetic variations control the transcriptome, which in turn affects e.g., the proteome (20), and metabolome (Figure 2, middle panel), our main focus will be pair-wise integrations linking genetic variation related to CAD risk to other down-stream omics layers such as epigenome, transcriptome, proteome or metabolome. Although multi-dimensional integrations have been widely used in the field of cancer research, their application in the context of CAD has so far been limited (22). Moreover, in addition to the autosomal genome, we will also consider the genetic variation in our “second genome”—the mitochondrial genome and its contribution to CAD.

## Integrating genetic variation and epigenome

Epigenomic signatures reflect various DNA modifications and may affect gene regulatory mechanisms that do not involve changes in the DNA sequence *per se*. Thereby, epigenomics may become a critical mediator of environmental influences and risk factors acting on the genome (20, 24). Three unique, but highly interrelated, epigenetic processes can be distinguished: DNA methylation, histone modifications (e.g., methylation, acetylation, phosphorylation, DP-ribosylation, and ubiquitination) and RNA-based mechanisms (e.g., microRNAs, long non-coding RNAs or lncRNAs, small interfering RNAs) (20, 24). Although, technically non-coding RNAs belong to the epigenome (20), we will discuss them in the next section, as the respective omics data are acquired via transcriptome profiling (RNA-seq).

**Figure 3:**
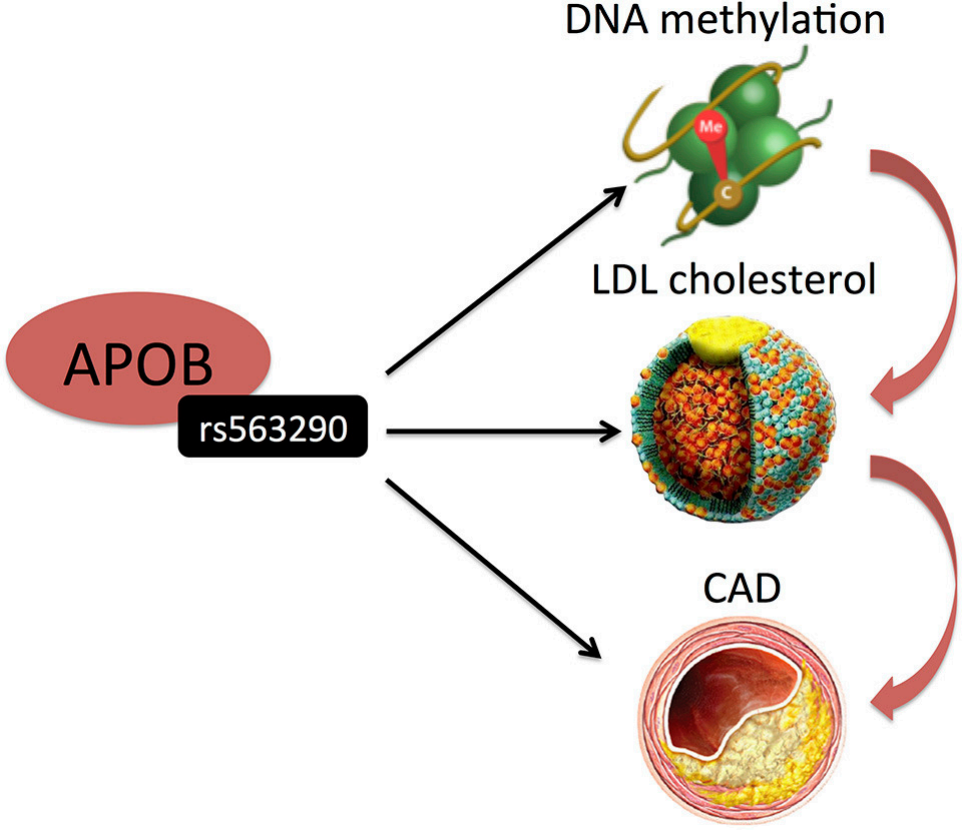
Hedman et al. (27) identified SNP (rs515135) in an intron of APOB to be associated with LDL-C. Its proxy was also associated with CAD. Interestingly, this SNP represents a cis-meQTL. Black arrows indicate association findings. Red arrows indicate the presumed functional cascade leading to CAD.

DNA methylation and histone modifications are the best understood of the epigenetic mechanisms thus far and have been widely suggested to regulate gene expression and affect CAD risk factors including atherosclerosis, inflammation, hypertension and diabetes (25). DNA methylation consists of the covalent methylation of the C5 position of cytosine residues, when they are followed by guanine residues (CpG dinucleotides). It is partly heritable but it is also a dynamic process related to environmental stimuli and life style factors (26). Hedman et al. (27) analyzed epigenetic changes associated with lipid concentrations and identified a number of meQTLs, enriched in signals from GWAS on lipid levels and CAD. For example, genome-wide significant variants (rs563290 and its proxies), associated with LDL cholesterol and CAD at APOB, were meQTLs for a LDL cholesterol-related differentially methylated locus (Table 1 and Figure 3).

**Table 1:** Genetic variation related to CAD/MI risk that has been associated with other down-stream omics layers such as transcriptome (mRNA, microRNAs and lncRNAs), epigenome, proteome or metabolome.

**Data type**	**Tissue**	**Phenotypic Trait**	**SNP**	**Omics-Marker**	**Refernces**
Transcriptome: mRNAs	Visceral abdominal fat	HDL cholesterol level	rs4148008	ABCAB/ABCA5	Franzén et al. (28)
			rs11869286	STARD3	
		Total cholesterol level	rs751557	EVI5	
			rs174546	TMEM258	
		LDL cholesterol level	rs12046679	PCSK9	
		CAD	rs892006	G3BP1	Foroughi Asl et al. (29)
			rs6908994	PSORSIC3	
			rs9930148	FLYWCH1	
	Internal mammary artery, atherosclerotic aortic root		rs7500448	CDH13	Nelson et al. (13)
	Blood	Blood pressure	Rs3184504	SH2B3,ALDH2,NAA25(cis) and IL8,TAGAP (trans)	Huan et al. (30)
Transcriptome: microRNAs	Circulating leukocytes, human coronary artery smooth muscle cells (HCASMC)	CAD	rs12190287	miR-224: TCF21	Miller et al. (31) Bastami et al. (32)
		The effect of diet on plasma lipid levels	rs13702	miR-410:LPL	Richardson et al. (33)
		CAD	rs989727(rs7808424)	miR-202-5p:ASZ1	Bastami et al. (34)
			rs41269915(rs2229238)	miR-485-5p:UBE2Q1	
			rs15563	hsa-miR-130a-5p:UBE2Z	Brænne et al. (16)
			rs3088442	hsa-miR-130a-5p:SLC22A3	
			rs2266788	hsa-miR-4722-5p:APOA5	
			rs72932707	hsa-miR-4722-5p:ICA1L	
		HDL,LDL, and total cholesterol,triglycerides	rs2370747(rs7115089	miR-100-5p,miR-125b-5p	Huan et al. (35)
		CAD	rs11042699 (rs6578985)	miR-483-3p-IGF2	
		Platelet count	rs4905998 rs(7149242)	miR-127-3P, miR-134, miR-370, miR-376a-3p, miR-382-5p, miR-431-5p, miR-433, miR-329, miR-409-3p, miR-494, miR-411-3p, miR-654-5p, miR-668, miR-543, miR-323a-3p, miR-337-3p	
		3p/5p ratio	rs13064131	miR-28:LPP	Civelek et al. (36)
Transcriptome:lnc RNAs	Internal mammary artery, atherosclerotic aortic root	CAD	rs1333045	FPKM1_group_33469_transcript_1	Ballantyne et al. (37)
		MI	rs1333049	FPKM1_group_33469_transcript_2	
		T2D	rs2383208	FPKM1_group_33469_transcript_6	
		Early MI	rs10757274	ANRIL	McPherson et al. (38)
Epigenome:DNA methylation		Hypertension	rs113460564, rs12443878, rs12444338, rs62040565, rs8060301	CDH13	Putku et al. (39)
		Diastolic blood pressure,serum high-density lipoprotein,high molecular weight adiponectin	rs8060301	Cg09415485(CDH13)	
		High molecular weight adiponectin	rs2239857, rs77068073	CDH13	
		Smoking(no assosciation)	rs75509302	cg23576855(AHRR)	
		LDL cholesterol level	rs563290 (rs515135, rs562338)	Cg05337441(APOB)	Hedman et al. (27)
Proteome	Blood plasma	CAD	rs12740374	Granulin(CELSR2/SORT1)	Chen et al. (40)
			rs867186	Protein C (PROCR)	Howson et al. (14)
			rs1050362	apolipoprotein L1 (DHX38)	
Metabolome	Blood plasma	CAD	rs715	CPS1,urea cycle metabolites,plasma glycine	Hartiala et al. (41)
	Blood plasma		rs10450989(USP3), rs2228513(HER-C1)rs930491,rs11827377(STIM1), rs3853422(SEL1L),rs1869075(F-BXO25),rs9591507,rs17573278,rs894840,rs9285184(SUGT1)	Circulating short-chain di-carboxylacylcarnitine(SC-DA)	Kraus et al. (42)
Multi-OMICS	Low HDL and inflammatory pathways		rs241437	TAP2	Laurila et al. (43)
			rs9272143	HLA-DRB1,HLA-DQA1	
Mitochondrial Genome	Blood	Hypertension	m.8701A>G	MT-ATP6	Zhu et al. (44)
		CAD	Haplogroup T		Kofler et al. (45)
			m.16189T>C		Mueller et al. (46)
			m.15927G>A		Jia et al. (47)

Furthermore, the CDH13 (T-cadherin) locus may present an interesting example in the context of epigenetics and CAD. Putku et al. (39) reported several genetic variants in the promoter of CDH13 as meQTLs in hypertension patients (Table 1), several of them being also associated with high molecular weight adiponectin, a known ligand for CDH13, the binding of which results in increased proliferation and migration of endothelial cells (39). Moreover, recently Nelson et al. (13) identified a genetic variant in the intron of CDH13, which affects expression of this gene in vascular tissues, and is genome-wide significantly associated with CAD (28) (Table 1). Interestingly, the expression levels of CDH13 and lncRNAs from the same locus showed positive correlations, suggesting a functional link, as lncRNAs are known to display correlations with the expression of their neighboring protein-coding target genes (48).

An exciting field of future research will be studies conducting parallel profiling of genetic variation with histone modifications and Hi-C and ChIA-PET-based chromatin contact maps to uncover local and distal histone quantitative trait loci (hQTLs) (49) in CAD patients.

Overall, considering the critical role of epigenetic modifications as a critical mediator of environmental influences on the genome (20, 24), we urgently need more investigations studying DNA methylation and other epigenetic modifications genome-wide and in large enough cohorts, ideally also elucidating the differences between tissues and cells in healthy vs. CAD patients. Moreover, this should be supplemented with careful documentation of multiple environmental and lifestyle factors over time, i.e., the envirome, as well as comprehensive clinical information to draw a link between the environment and CAD.

## Integrating genetic variation and transcriptome

Transcriptomics reflect genome-wide measures of RNA levels, both protein-coding RNA as well as the non-coding RNAs (i.e., microRNAs, lncRNAs, and small interfering RNAs) under specific conditions or in a specific cell. Moreover, the transcript levels are examined both qualitatively (i.e., which transcripts are present, identification of novel transcripts, splice sites, and RNA editing sites) and quantitatively (quantification of transcript abundance) (21).

### Protein-coding RNAs

Parallel assessments of genetic variation and transcriptome profiles across disease-relevant tissues, i.e., via mapping expression quantitative trait loci (eQTLs) to identify susceptibility genes (mainly protein-coding), has been the most commonly applied approach (28, 29, 50–52). Björkegren et al. have performed a number of integrative network analysis, linking CAD risk variants and transcriptome data in seven disease-relevant vascular and metabolic tissues, collected from up to 600 CAD patients during coronary artery bypass surgery (28, 29, 53, 54). From these investigations, visceral abdominal fat has emerged as an important gene-regulatory site for blood lipids. Several risk SNPs for HDL-, LDL-, and total cholesterol levels, as well as for CAD demonstrated significant eQTL effects in visceral abdominal fat (28, 29).

**Figure 4:**
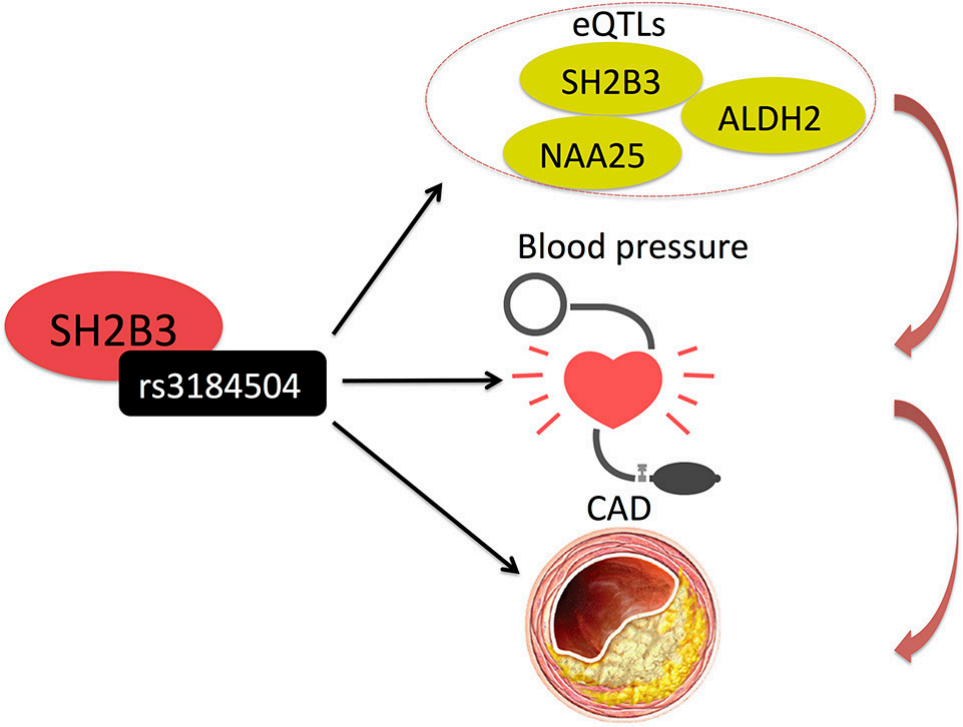
Huan et al. (30) uncovered a blood pressure associated SNP (rs3184504) in SH2B3, which also associates with the expression (eQTL) of several genes, including SH2B3 itself, in the genetically inferred causal blood pressure gene sets. Rs3184504 has been previously also associated with CAD risk. (9) Black arrows indicate association findings. Red arrows indicate the presumed functional cascade leading to CAD.

Huan et al. (30) also used integrative analysis to investigate the molecular mechanisms of blood pressure regulation and identified a blood pressure associated SNP (rs3184504) in SH2B3, also associated with the expression (eQTL) of several genes, including SH2B3, in the genetically inferred causal blood pressure gene sets (Table 1 and Figure 4). Some of these genes were also perturbed in Sh2b3^−/−^ mice, demonstrating blood pressure-related phenotype (30). Rs3184504 has been previously also associated with CAD risk (9).

Much less investigated are non-coding RNA transcripts, such as micro-RNAs (miRNAs) and long non-coding RNAs (lncRNAs). Recent evidence suggests that at least some of these may play a role in CAD (55–58). Although, technically non-coding RNAs belong to the epigenome (20), we will discuss them in this section, as the respective omics data are acquired via transcriptome profiling (RNA-seq).

### Micro RNAs

MiRNAs are involved in the transcriptional control of all main cell types participating in atherosclerosis progression, including endothelial cells, vascular smooth muscle cells, and macrophages (32, 59). Several studies have investigated the differential expression patterns of miRNAs in plasma/serum, microparticles, whole blood, platelets, blood mononuclear intimal, and endothelial progenitor cells in CAD vs. non-CAD patients, as summarized by Malik et al. (60). In majority of cases, up-regulation of different miRNA in CAD patients was observed (60). Moreover, growing body of evidence suggests that genetic variations in the miRNA targetome may lead to major deleterious outcomes (61, 62). For example, Miller et al. (31) have shown that an established CAD risk variant (rs12190287) resides in the 3′ untranslated region of a transcription factor TCF21 and alters the seed binding sequence for miR-224. Moreover, allelic imbalance studies in circulating leukocytes and human coronary artery smooth muscle cells have demonstrated a significant imbalance of the TCF21 transcript levels, which correlated with genotype at rs12190287, consistent with this variant contributing to allele-specific expression differences (31). Richardson et al. (33) have reported that a variant (rs13702) in the 3'-UTR of lipoprotein lipase (LPL) disrupts the binding of miR-410 and modulates the effect of diet on plasma lipid levels (33). Recently, Bastami et al. (34) performed a more systematic computational screening, by mapping the established CAD risk variants to the miRNA targetome, identifying several links between SNPs and miRNAs (Table 1; https://www.ebi.ac.uk/gwas/). In a recent study from our group (16), we also mapped CAD risk variants from the CARDIoGRAMplusC4D GWAS meta-analyses (9), to 3′ UTR regions of genes to assess their overlaps with predicted target miRNA binding sites. Interestingly, the 3′ UTR region of MRAS was predicted to be targeted by 29 miRNAs and 23 miRNAs were predicted to bind more than one candidate CAD gene (Table 1). Thus far, there have been relatively few studies investigating genome-wide miRNA eQTLs (miR-eQTLs). Huan et al. (35) identified a genetic variant (rs2370747) associated with miR-100-5p and miR-125b-5p expression, a proxy SNP of which was also associated with lipid traits (HDL-, LDL-, and total cholesterol as well as triglycerides). Moreover, it was found that both miRNAs were also differentially expressed in relation to HDL cholesterol (35).

**Figure 5:**
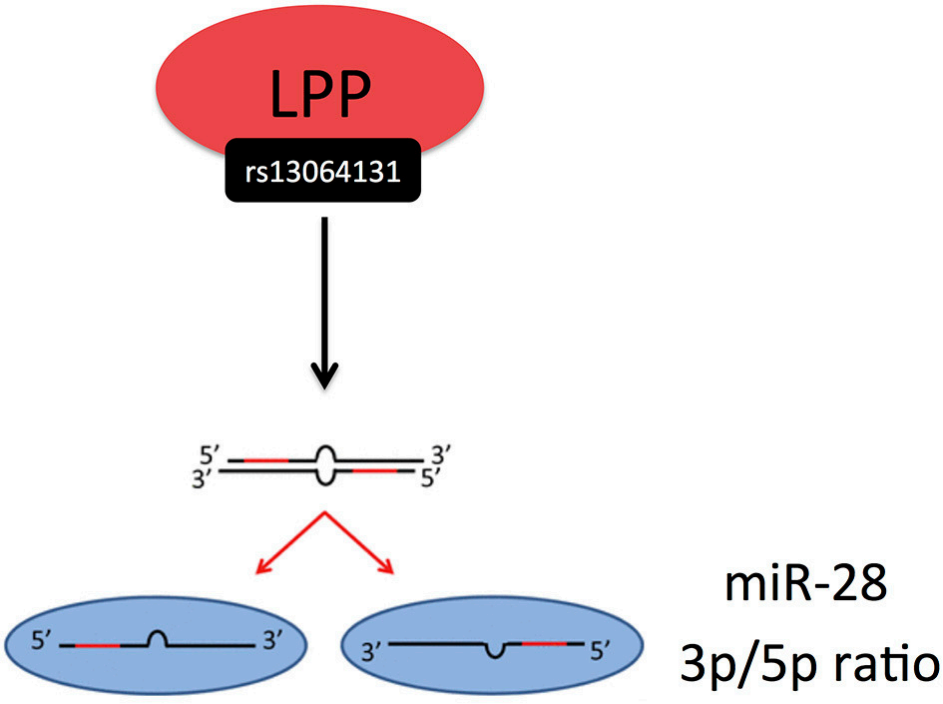
Civelek et al. (36) demonstrated a significant association of the SNP rs13064131 with the 3p/5p ratio of miR-28, encoded from the LPP gene. The miRNA processing and strand selection was adapted from (65).

Civelek et al. (36) examined the genetic regulation of human adipose miRNA expression and its consequences for metabolic traits. Interestingly, this study showed, how genetic variation might influence the processing of miRNAs, i.e., the ratio of miRNA expression from the 3p and 5p arms. It is known that a miRNA precursor can give rise to two mature miRNAs from the 3p and 5p arm, one of which usually having higher expression than the other. The 3p/5p ratios of several miRNAs have been shown to be significantly different among various healthy tissues (63) and altered in pathological conditions compared with healthy controls (64). Civelek et al. demonstrated a significant association of the SNP rs13064131 with the 3p/5p ratio of miR-28, encoded from the LPP gene (Figure 5) (36). However, the SNP was not associated with the expression levels of the LPP transcript itself or with the abundance of miR-28-3p or miR-28-5p, suggesting that its effect on the 3p/5p ratio may be independent of transcription, possibly via degradation or stabilization mechanisms.

### Long non-coding RNAs

**Figure 6:**
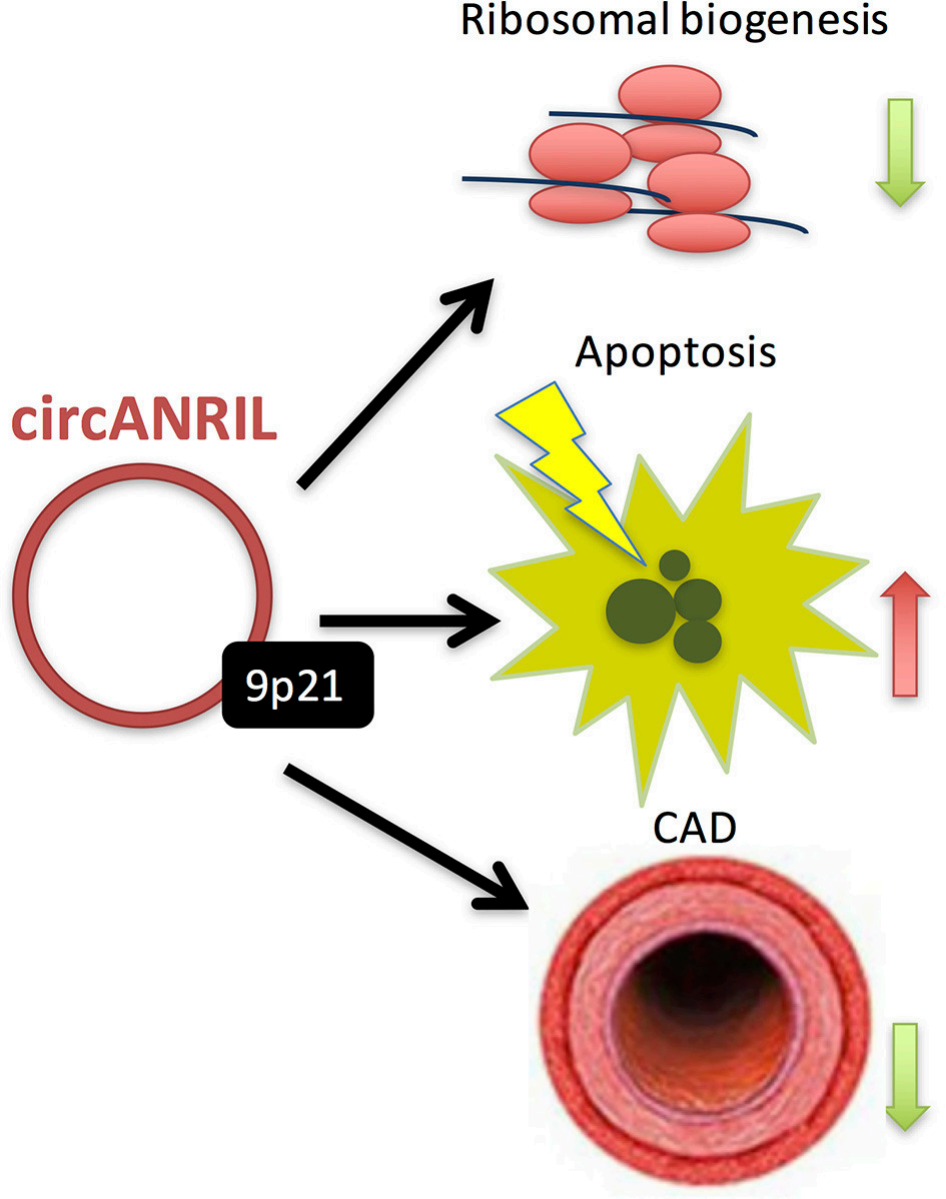
Recently, Holdt et al. (69) demonstrated that circANRIL regulates the maturation of precursor ribosomal RNA (pre-rRNA), by this impairing ribosome biogenesis and inducing nucleolar stress and apoptosis in vascular smooth muscle cells and macrophages. Moreover, carriers of the CAD-protective haplotype at 9p21 showed significantly increased expression of circANRIL.

The recent discovery of an extensive catalog of lncRNAs—i.e., long RNA transcripts that do not code for proteins—has opened a new perspective on the importance of the RNA-based mechanisms in gene regulation (24). LncRNAs are emerging as important regulators of various cellular processes, with many possible implications in cardiovascular disease pathophysiology (57, 58). In fact, the most prominent CAD risk locus at Chr9p21 (66, 67) harbors the lncRNA—ANRIL (Antisense Non-coding RNA in the INK4 Locus, CDKN2B antisense RNA). From these, rs10757274 is the strongest genetic predictor of early MI and is not associated with established CAD risk factors such as lipoproteins or hypertension, making ANRIL a key candidate (38). Interestingly, ANRIL is found both as a linear lncRNA (linANRIL), the transcript levels of which are known to positively correlate with disease severity (68), and is also capable of forming RNA circles (circANRIL) (69). Recently, Holdt et al. (69) demonstrated that circANRIL regulates the maturation of precursor ribosomal RNA (pre-rRNA), by this impairing ribosome biogenesis and inducing nucleolar stress and apoptosis in vascular smooth muscle cells and macrophages (Figure 6). Carriers of the CAD-protective haplotype at 9p21 showed significantly increased expression of circANRIL (69).

Currently, there have not been many large-scale studies on lncRNAs in the context of CAD, though. Ballantyne et al. (37) recently conducted a genome-wide interrogation of long intergenic non-coding RNAs (lincRNAs) that associate with cardiometabolic traits in GWAS, including CAD and also identified a number of CAD/MI and type 2 diabetes associated SNPs at Chr9p21 that overlapped lincRNA transcripts (Table 1) (37). In STARNET (28), 5.4% of the identified cis-expression quantitative trait loci (eQTLs) were related to the expression of lncRNAs, however these have not been further explored, so far. Overall, more studies focusing on non-coding RNAs in different CAD relevant tissues in large enough cohorts will be required to yield insights into the possible functional roles of this portion of transcriptome and its genetic determinants, in healthy and disease states. Moreover, considering that lncRNAs are generally found to be more lowly-expressed, sufficient depth of coverage for RNA-seq experiments will need to be guaranteed (21).

## Integrating genetic variation and proteome

Proteomics uses high-throughput approaches (mainly MS-based) to quantify protein abundance, post-translational modifications and interactions (e.g., using phage display and yeast two-hybrid assays) in a tissue, cell or fluid compartment, such as plasma or urine (21). Considering that the transcriptome is not linearly proportional to proteome, that proteins are the biomolecules that execute cellular functions, and that many human diseases ultimately result from alterations in the proteome (70), such studies are urgently needed to facilitate the explorations of CAD etiology. However, proteome studies are still rare in relation to CAD, mostly due to the complex methodology involved. There have been some investigations in the past few years, aiming at characterizing the proteomes of several CAD-related tissues and cell types, including human arterial smooth muscle cells (71), platelets (72), as well as body fluids such as urine (73).

**Figure 7:**
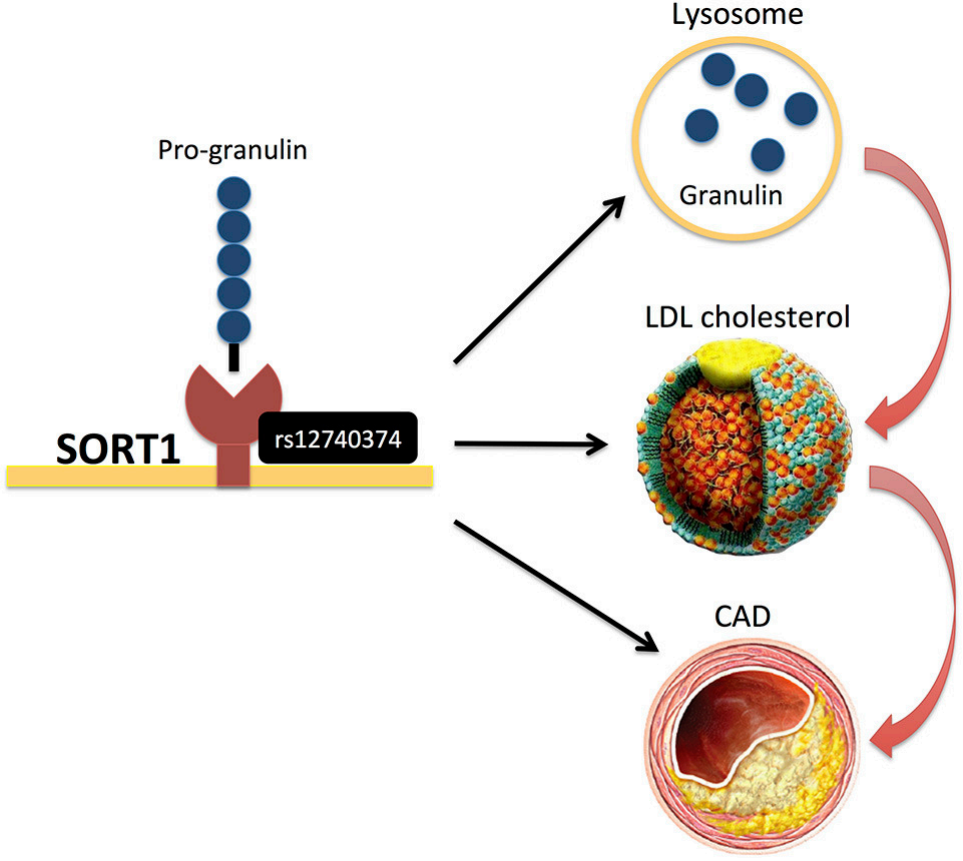
rs12740374 at the CELSR2/SORT1 locus, (40) a variant associated with lipids and CAD, was recently found to display pQTL effects on plasma granulin levels, and pro-granulin is known to bind to SORT1. More recently, it was also demonstrated that progranulin is involved in lysosomal homeostasis and lipid metabolism (74).

Only few studies (14, 40) have analyzed genetic variants that modify protein levels, i.e., the so-called protein quantitative trait loci (pQTLs) (Table 1). Chen et al. (40) assayed a pre-selected set of plasma proteins, identifying several pQTLs that overlapped with CAD risk SNPs and also explained a substantial proportion of inter-individual variation in protein abundance. For example, rs12740374 at the CELSR2/SORT1 locus, a variant associated with lipids and CAD, explained 15% of inter-individual variation in plasma granulin levels (Figure 7). Interestingly, progranulin binds to SORT1 and *Sort1* knockout mice show markedly elevated levels of progranulin (40). Recently, it was also demonstrated that progranulin is involved in lysosomal homeostasis and lipid metabolism (74).

As the proteomics technologies improve over time (21), more genome-wide investigations of CAD-related alterations in proteome and also phosphorpoteome in increasing numbers of disease relevant tissues are expected to be conducted in the near future. However, as proteins are more sensitive to their environment (21), caution will have to be taken during sample preparation steps to obtain accurate and reproducible results.

## Integrating genetic variation and metabolome

**Figure 8:**
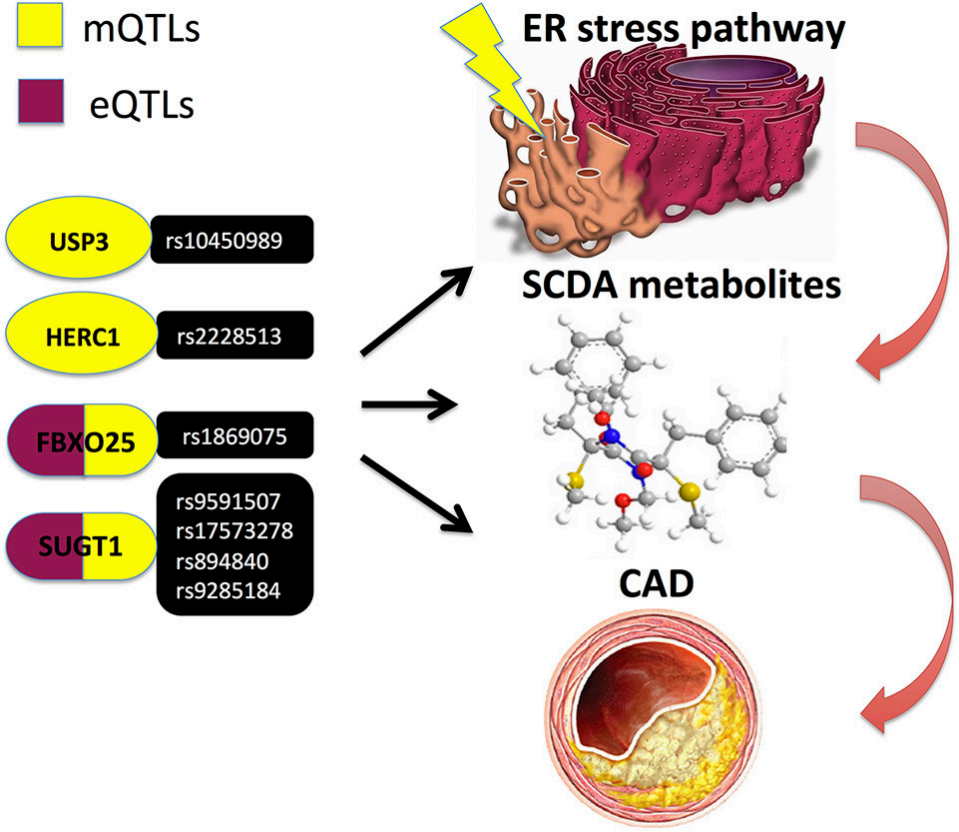
Kraus et al. (42) performed a pathway-level integrative analyses and observed associations of circulating short-chain dicarboxylacylcarnitine (SCDA) with variants in ER stress genes, whereof several genetic variants in FBXO25 and SUGT1 genes also demonstrated evidence of cis-regulation in expression quantitative trait loci (eQTL) analyses and independently predicted CAD events.

An important additional functional layer in mutli-omics data integration is the metabolome, as it represents an integrated state of all genetic, epigenetic and environmental factors, thus providing a link between genotype and phenotype (75). Metabolomics is an omics field that systematically identifies and quantifies multiple small molecule (typically < 1,500 Daltons) types, such as amino acids, fatty acids, carbohydrates and biochemical intermediates, i.e., metabolites (21). A plethora of metabolites in blood and urine have been associated with CAD and subsequent cardiovascular events (76–79) and have been demonstrated as promising biomarkers discriminating CAD vs. non-CAD subjects (78), as well as between thrombotic MI and stable CAD cases (80). Kraus et al. (42) recently identified several genetic loci demonstrating associations with blood plasma metabolites (i.e., metabolomic quantitative trait loci; mQTLs), the strongest findings being for the circulating short-chain dicarboxylacylcarnitine (SCDA) metabolite levels with variants in genes that regulate components of endoplasmic reticulum (ER) stress (Table 1 and Figure 8) (42).

Besides blood and urine, metabolomic profiles of vascular and metabolomic tissues such as subcutaneous fat will need to be generated, ideally in conjunction with other omics layer data. Especially, gut microbiome would be of utmost interest, considering the close link between the two (81).

However, of note, metabolic profiles are even more prone to variability affected by sample preparation and storage conditions, as well as by several other factors including patient heterogeneity (21). Hence, the required sample size has to be carefully considered, to inspire confidence in the generated results.

## Integrating genetic variation and microbiome

Microbiomics investigates all the microorganisms of a given community, including bacteria, viruses, and fungi, collectively known as the microbiota (and their genes constituting the microbiome) (21). The human microbiome is enormously complex and there are substantial variations in microbiota composition between individuals resulting from seed during birth and development, diet and other environmental factors, drugs and age (21). Thousands of different bacterial species make up the human microbiomes, from which there is a small number of abundant species and a large number of rare or low abundance species, the differential functions of which remain poorly understood (82). Currently, several large scale initiatives are emerging including the American Gut Project http://americangut.org/ and the British Gut Project http://britishgut.org/, which are expected to produce a rich collection of anonymised human gut samples and lifestyle information for medical researchers.

Gut microbiome has emerged as another rich source of information and as a possible new player contributing to the CAD/MI pathogenesis (82–84). It has long been known that bacteria activate inflammatory pathways, and recent data demonstrate that the gut microbiome may also affect lipid metabolism and influences the development of obesity and atherosclerosis (84), suggesting that gut microbiota could be used as a diagnostic marker for CAD (85). The most investigated is the association between gut microbiota and fasting plasma trimethylamine N-oxide (TMAO) levels, a gut microbiota-dependent metabolite, previously also associated with CAD and stroke (81, 86). Org et al. (81) demonstrated that certain blood plasma metabolites strongly correlated with gut microbial community structure and that some of these correlations may be specific for the pre-diabetic state. LeChatelier et al. (84) used qunatitative gut microbiome information to distinguish between individuals with “high bacterial richness” and “low bacterial richness,” were the latter were characterized by increased adiposity, insulin resistance and dyslipidemia in addition to a more pronounced inflammatory phenotype. Le Chatelier Fu et al. (84) and Fu et al. (87) reported that gut microbiota richness and diversity were negatively correlated with triglycerides and positively correlated with HDL levels, however this effect was independent of age, sex and host genetics. So far, genome-wide mapping of the so-called microbiome quantitative trait loci (mbQTLs) (88) in the context of CAD has not been performed and is definitely next in line, ideally in conjunction with comprehensive profiling of metabolome in several tissues and body fluids in large enough cohorts.

## Integrating genetic variation and multiple omics datasets

An integrative analysis of genetic variation and transcriptome with additional high-throughput measurements may greatly improve the predictive power of disease networks. Zhu et al. (89) However, the number of studies conducting multi-omics integrations in the context of CAD is limited so far. Miller et al. (90) integrated genetic variation with investigations of chromatin state, enhancer activity and TF binding in human coronary artery smooth muscle cells and demonstrated, for example, that one of the lead candidate variants, rs17293632, located within an intergenic region of the SMAD3 gene, overlaps an open chromatin region. Moreover, it was observed that the major risk C allele was more associated with open chromatin and resided in a canonical AP-1 motif, which was effectively destroyed by the minor protective T allele. Preferential AP-1 binding to the risk C allele was experimentally validated using allele-specific ChIP analyses. Miller et al. (90) and Kraus et al. (42) performed a pathway-level integrative analyses, linking genetics, epigenetics, transcriptomics, and metabolomics profiles and implicating the ubiquitin proteasome system in cardiovascular disease pathogenesis. This study observed associations of circulating short-chain dicarboxylacylcarnitine (SCDA) with variants in ER stress genes, whereof several genetic variants (Table 1 and Figure 8) in FBXO25 and SUGT1 genes also demonstrated evidence of cis-regulation in expression quantitative trait loci (eQTL) analyses and independently predicted CAD events (42). Moreover, two other genes from the same ER stress pathway—BRSK2 and HOOK2—were identified as differentially methylated, when comparing individuals with high and low SCDA levels. Subsequently, experimental validation using culture of human kidney cells in the presence of levels of fatty acids found in individuals with cardiometabolic disease, demonstrated induced accumulation of SCDA metabolites in parallel with increases in the ER stress marker BiP (42).

Shu et al. (20) investigated shared genetic regulatory networks for CAD and type 2 diabetes (T2D) and their key intervening drivers in multiple populations of diverse ethnicities by performing an integrative analysis of five multi-ethnic GWAS for CAD and T2D, eQTLs, ENCODE, as well as tissue-specific gene network models (both co-expression and graphical models) from disease-relevant tissues. This study identified pathways regulating the metabolism of lipids, glucose and branched-chain amino acids, as well as pathways governing oxidation, extracellular matrix and immune response as shared pathogenic processes for both diseases and identified 15 key drivers including HMGCR, CAV1, IGF1, and PCOLCE, whose network neighbors collectively accounted for ~35% of known GWAS hits for CAD and 22% for T2D (20). Laurila et al. (43) applied a combined approach using both QTLs and canonical pathway analysis to link genomics and transcriptome analysis from the subcutaneous adipose tissue and plasma HDL lipidomics profiling, highlighting change in HDL particle quality toward putatively more inflammatory and less atheroprotective phenotype in subjects with low HDL, due to their reduced antioxidative capacity. Within the HLA region, this study found two significant, dose-dependent cis-eQTL associations with low HDL and inflammatory pathways: rs241437 in the intron of TAP2 and rs9272143 between HLA-DRB1 and HLA-DQA1, the latter also being associated with down-regulation of antioxidative pathways in HDL particles (43).

The application of multi-omics integrations in the field of CAD has so far been limited (22). Obviously, one of the main reasons for this is the current lack of appropriate data in large enough cohorts. However, considering the great promise such studies hold for precision medicine, it is expected that parallel measurements on multiple omics layers will be rapidly collected during the next couple of years, allowing also a comprehensive comparison, validation and improvement of the existing computational integration methods.

## Mitochondrial genetic variation and downstream omics datasets

**Figure 9:**
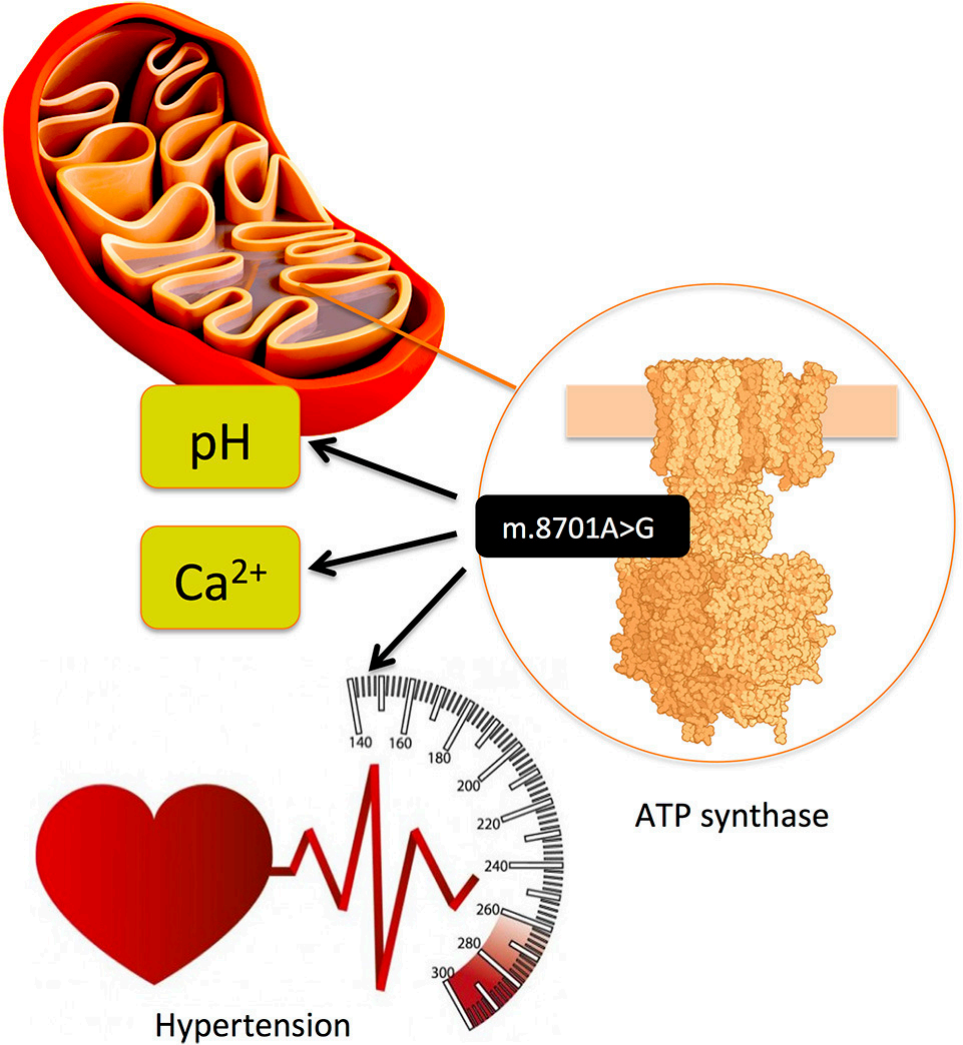
Mitochondrial variant m.8701A>G is located in MT-ATP6 (ATP synthase/complex V F0 subunit 6) gene, which is part of the ATP synthase enzyme, responsible for the final step of oxidative phosphorylation and has been associated with hypertension. (44) On the functional level, using transmitochondrial hybrid cells (cybrids), it has been shown that it alters mitochondrial matrix pH and intracellular calcium dynamics (92).

Dysfunction of mitochondria has been increasingly associated with obesity-related cardiometabolic diseases and CAD (91). Thus, genetic variation in the mitochondrial DNA (mtDNA), which codes for the 37 OXPHOS genes as well as further >1000 nuclear-coded genes imported into mitochondria constituting essential components for their proper functioning, needs exploration for a better understanding of CAD genetics. The mitochondrial haplogroup T (45) and mtDNA variants m.16189T>C (46) and m.15927G>A (47) have been associated with CAD in different ethnic groups. Another mitochondrial variant, m.8701A>G, has been associated with hypertension (44). This variant is located in MT-ATP6 (ATP synthase/complex V F0 subunit 6) gene, which is part of the ATP synthase enzyme, responsible for the final step of oxidative phosphorylation, and, on the functional level, using transmitochondrial hybrid cells (cybrids), it has been shown that it alters mitochondrial matrix pH and intracellular calcium dynamics (Figure 9) (92).

Similarly, other mitochondria-related omics data investigations could be of interest in the context of CAD, as Baccarelli et al. (93) reported that ATP synthesis genes including protein-encoding cytochrome c oxidase genes (MT-CO1, MT-CO2, and MT-CO3) and MT-TL1 were hypermethylated in platelets of CAD cases as compared to healthy controls (93). Using eQTLs in seven CAD relevant vascular and metabolic tissues (53) in conjunction with established CAD risk loci from GWAS (9) and time-resolved transcriptome data in the aortic arch in mice with reversible hypercholesterolemia (94, 95) we recently demonstrated a massive down-regulation of nuclear-encoded mitochondrial genes (96), specifically at the time of rapid atherosclerotic lesion expansion and foam cell formation, which was largely reversible by genetically lowering plasma cholesterol. Both mitochondrial signature genes were supported as causal for CAD in humans, as eQTLs representing their genes significantly overlapped with disease risk SNPs. In line with this, the STARNET (28) study recently examined mitochondrial (i.e., mtDNA-derived) gene expression and a markedly lower expression of mitochondrial genes in the atherosclerotic aortic arterial wall as compared to non-atherosclerotic arterial wall.

Furthermore, genetic variation of mitochondrial metabolome has remained largely unexplored. Hartiala et al. (41) searched for genetic factors associated with plasma betaine levels and determined their effect on CAD risk. This resulted in the identification of two significantly associated loci on chromosomes 2q34 and 5q14.1. The lead variant on 2q24—rs715—localized to carbamoyl-phosphate synthase 1 (CPS1), which encodes a mitochondrial enzyme that catalyzes the first committed reaction and rate-limiting step in the urea cycle. Rs715 was also significantly associated with decreased levels of urea cycle metabolites and increased plasma glycine levels. Finally, rs715 yielded a strikingly significant and protective association with decreased risk of CAD in women (41).

Finally, in recent years, it has become increasingly evident that the gut microbiome produces metabolites that influence mitochondrial function and biogenesis (97), hence the ancestral gut microbiome-mitochondrion connection and its relation to CAD might need to be explored in the near future, as well.

Resent progress in next-generation sequencing (NGS) techniques has set a scene for a second “gold rush” in mitochondrial genomics and mtDNAs are presently the most sequenced type of eukaryotic chromosome (98). At the same time, multi-omics investigations in mitochondria, mapping the genomes, transcriptomes, proteomes, and metabolomes in parallel, apart from yeast (99) have not been conducted yet. Hence, although, mitochondrial dysfunction has been associated with many human diseases, the respective proteins and pathways are not well-characterized (99), presenting an exciting future field of investigation, especially considering the fact that mitochondria play a key role in plasticity and adaptation to environmental change, including adaptation to physiological stress (100).

## Conclusions and future directions

Given that CAD like other common complex disorders develops over time and involves both genetics and environment, full mechanistic insight will require coordinated sets of several-omics data at multiple time points, collected from many disease relevant tissues and body fluids in large enough cohorts (20, 21). Environmental risk factors can interact with the genome and perturb the epigenome to further modulate the transcriptome and proteome (20). Therefore, comprehensive monitoring and careful documentation of multiple environmental and lifestyle factors over time, i.e., the envirome, will be indispensable to yield significant insights into the complex etiology of CAD. Moreover, imaging and electronic health record data also will need to be considered. As more-omics and other data are generated, novel methods for efficient data integration, modeling, visualization and interpretation will be urgently needed to efficiently cope with this multi-dimensional data (101), and translate it into actionable precision medicine tools. Although, there has been major progresses in the development of multidimensional data integration algorithms and tools, the field is still in its infancy and the flexibility, effectiveness and robustness of data integration to extract biological insights is still restricted, especially when clinical outcomes (e.g., stable CAD vs. MI) need to be modeled (22, 101). In addition we still face a number of technical challenges related to patient sampling and profiling. For example, as already recognized by Hasin et al. and others (20, 21) human studies are often affected by various confounding factors, which are difficult or even impossible to control for (e.g., diet and medications). Clearly, also the available sample size will play an important role for the multi-omics approach to produce meaningful insights into CAD (21) and allow the generation of reliable prediction models for more efficient design of therapeutics, tailored to individual needs. According to Hasin et al. an underpowered study may not only miss true signals, but is also more likely to produce false positive results (21). Furthermore, already before and during data collection, careful attention has to be paid to data analysis requirements, e.g., sufficient depth of coverage for RNA-seq experiments (21).

## Author contributions

BV and HS drafted and edited the manuscript.

### Conflict of interest statement

The authors declare that the research was conducted in the absence of any commercial or financial relationships that could be construed as a potential conflict of interest.

## References

[1] Fernández-Friera L Fuster V López-Melgar B Oliva B García-Ruiz JM Mendiguren J. Normal LDL-cholesterol levels are associated with subclinical atherosclerosis in the absence of risk factors. J Am Coll Cardiol. (2017) 70:2979–91. 10.1016/j.jacc.2017.10.024 29241485

[2] Kessler T Vilne B Schunkert H. The impact of genome-wide association studies on the pathophysiology and therapy of cardiovascular disease. EMBO Mol Med. (2016) 8:688–701. 10.15252/emmm.201506174 27189168 PMC4931285

[3] Ference BA Ginsberg HN Graham I Ray KK Packard CJ Bruckert E. Low-density lipoproteins cause atherosclerotic cardiovascular disease. 1. Evidence from genetic, epidemiologic, and clinical studies. A consensus statement from the European Atherosclerosis Society Consensus Panel. Eur Heart J. (2017) 38:2459–72. 10.1093/eurheartj/ehx144 28444290 PMC5837225

[4] Erdmann J Grosshennig A Braund PS König IR Hengstenberg C Hall AS. New susceptibility locus for coronary artery disease on chromosome 3q22.3. Nat Genet. (2009) 41:280–2. 10.1038/ng.307 19198612 PMC2695543

[5] Erdmann J Willenborg C Nahrstaedt J Preuss M König IR Baumert J. Genome-wide association study identifies a new locus for coronary artery disease on chromosome 10p11.23. Eur Heart J. (2011) 32:158–68. 10.1093/eurheartj/ehq405 21088011

[6] Schunkert H König IR Kathiresan S Reilly MP Assimes TL Holm H. Large-scale association analysis identifies 13 new susceptibility loci for coronary artery disease. Nat Genet. (2011) 43:333–8. 10.1038/ng.784 21378990 PMC3119261

[7] Charchar FJ Bloomer LD Barnes TA Cowley MJ Nelson CP Wang Y. Inheritance of coronary artery disease in men: an analysis of the role of the Y chromosome. Lancet (2012) 379:915–22. 10.1016/S0140-6736(11)61453-0 22325189 PMC3314981

[8] Erdmann J Stark K Esslinger UB Rumpf PM Koesling D de Wit C. Dysfunctional nitric oxide signalling increases risk of myocardial infarction. Nature (2013) 504:432–6. 10.1038/nature12722 24213632

[9] CARDIoGRAMplusC4D Consortium Deloukas P Kanoni S Willenborg C Farrall M Assimes TL Large-scale association analysis identifies new risk loci for coronary artery disease. Nat Genet. (2013) 45:25–33. 10.1038/ng.2480 23202125 PMC3679547

[10] Nikpay M Goel A Won HH Hall LM Willenborg C Kanoni S. A comprehensive 1,000 Genomes-based genome-wide association meta-analysis of coronary artery disease. Nat Genet. (2015) 47:1121–30. 10.1038/ng.3396 26343387 PMC4589895

[11] Myocardial Infarction Genetics and CARDIoGRAM Exome Consortia Investigators Stitziel NO Stirrups KE Masca NG Erdmann J Ferrario PG Coding Variation in ANGPTL4, LPL, and SVEP1 and the risk of coronary disease. N Eng J Med. (2016) 374:1134–44. 10.1056/NEJMoa1507652 PMC4850838 26934567

[12] Webb TR Erdmann J Stirrups KE Stitziel NO Masca NG Jansen H. Systematic evaluation of peiotropy identifies 6 further loci associated with coronary artery disease. J Am Coll Cardiol. (2017) 69:823–36. 10.1016/j.jacc.2016.11.056 28209224 PMC5314135

[13] Nelson CP Goel A Butterworth AS Kanoni S Webb TR Marouli E. Association analyses based on false discovery rate implicate new loci for coronary artery disease. Nat Genet. (2017) 49:1385–91. 10.1038/ng.3913 28714975

[14] Howson J.MM Zhao W Barnes DR Ho WK Young R Paul DS. Fifteen new risk loci for coronary artery disease highlight arterial-wall-specific mechanisms. Nat Genet. (2017) 49:113–9. 10.1038/ng.3874 28530674 PMC5555387

[15] van der Harst P Verweij N The identification of 64 novel genetic loci provides an expanded view on the genetic architecture of coronary artery disease. Circ Res. (2017) 122:433–43. 10.1161/CIRCRESAHA.117.312086 29212778 PMC5805277

[16] Brænne I Civelek M Vilne B Di Narzo A Johnson AD Zhao Y. Prediction of causal candidate genes in coronary artery disease loci. Arterioscler Thromb Vasc Biol. (2015) 35:2207–17. 10.1161/ATVBAHA.115.306108 26293461 PMC4583353

[17] Ritchie MD Holzinger ER Li R Pendergrass SA Kim D. Methods of integrating data to uncover genotype-phenotype interactions. Nat Rev Genet. (2015) 16:85–97. 10.1038/nrg3868 25582081

[18] Hartiala J Schwartzman WS Gabbay J Ghazalpour A Bennett BJ Allayee H. The genetic architecture of coronary artery disease: current knowledge and future opportunities. Curr Atheroscler Rep. (2017) 19:6. 10.1007/s11883-017-0641-6 28130654 PMC5783155

[19] Manzoni C Kia DA Vandrovcova J Hardy J Wood NW Lewis PA. Genome, transcriptome and proteome: the rise of omics data and their integration in biomedical sciences. Brief Bioinformat. (2016) 19:286–302. 10.1093/bib/bbw114 27881428 PMC6018996

[20] Arneson D Yang X Shu L Bioinformatics principles for deciphering cardiovascular diseases. Encyclop Cardiovasc Res Med. (2018) 1:273–92. 10.1016/B978-0-12-801238-3.99576-X

[21] Hasin Y Seldin M Lusis A. Multi-omics approaches to disease. Genome Biology (2017) 18:83. 10.1186/s13059-017-1215-1 28476144 PMC5418815

[22] Arneson D Shu L Tsai B Barrere-Cain R Sun C Yang X. Multidimensional integrative genomics approaches to dissecting cardiovascular disease. Front Cardiovasc Med. (2017) 4:8. 10.3389/fcvm.2017.00008 28289683 PMC5327355

[23] Erdmann J Kessler T Munoz Venegas L Schunkert H. A decade of genome-wide association studies for coronary artery disease: the challenges ahead. Cardiovasc Res. (2018) 114:1241–57. 10.1093/cvr/cvy084 29617720

[24] Turgeon PJ Sukumar AN Marsden PA. Epigenetics of cardiovascular disease - a new “Beat” in coronary artery disease. Med Epigenet. (2014) 2:37–52. 10.1159/000360766 25408699 PMC4232955

[25] Muka T Koromani F Portilla E O'Connor A Bramer WM Troup J. The role of epigenetic modifications in cardiovascular disease: a systematic review. Int J Cardiol. (2016) 212:174–83. 10.1016/j.ijcard.2016.03.062 27038728

[26] Fernández-Sanlés A Sayols-Baixeras S Subirana I Degano IR Elosua R. Association between DNA methylation and coronary heart disease or other atherosclerotic events: a systematic review. Atherosclerosis (2017) 263:325–33. 10.1016/j.atherosclerosis.2017.05.022 28577936

[27] Hedman ÅK Mendelson MM Marioni RE Gustafsson S Joehanes R Irvin MR. Epigenetic patterns in blood associated with lipid traits predict incident coronary heart disease events and are enriched for results from genome-wide association studies. Circulation (2017) 10:e001487 10.1161/CIRCGENETICS.116.001487 28213390 PMC5331877

[28] Franzén O Ermel R Cohain A Akers NK Di Narzo A Talukdar HA Cardiometabolic risk loci share downstream cis- and trans-gene regulation across tissues and diseases. Science (2016) 353:827–30. 10.1126/science.aad6970 27540175 PMC5534139

[29] Foroughi Asl H Talukdar HA Kindt AS Jain RK Ermel R Ruusalepp A Expression quantitative trait loci acting across multiple tissues are enriched in inherited risk for coronary artery disease. Circ Cardiovasc Genet. (2015) 8:305–15. 10.1161/CIRCGENETICS.114.000640 25578447

[30] Huan T Meng Q Saleh MA Norlander AE Joehanes R Zhu J. Integrative network analysis reveals molecular mechanisms of blood pressure regulation. Mol Syst Biol. (2015) 11:799. 10.15252/msb.20145399 25882670 PMC4422556

[31] Miller CL Haas U Diaz R Leeper NJ Kundu RK Patlolla B. Coronary heart disease-associated variation in TCF21 disrupts a Mir-224 binding site and miRNA-mediated regulation. PLoS Genet. (2014) 10:e1004263. 10.1371/journal.pgen.1004263 24676100 PMC3967965

[32] Bastami M Ghaderian SM Omrani MD Mirfakhraie R Vakili H Parsa SA. MiRNA-related polymorphisms in MiR-146a and TCF21 are associated with increased susceptibility to coronary artery disease in an Iranian population. Genet Test Mol Biomark. (2016) 20:241–8. 10.1089/gtmb.2015.0253 26909569

[33] Richardson K Nettleton JA Rotllan N Tanaka T Smith CE Lai CQ. Gain-of-function lipoprotein lipase variant rs13702 modulates lipid traits through disruption of a microRNA-410 seed site. Am J Hum Genet. (2013) 92:5–14. 10.1016/j.ajhg.2012.10.020 23246289 PMC3542456

[34] Bastami M Nariman-Saleh-Fam Z Saadatian Z Nariman-Saleh-Fam L Omrani MD Ghaderian SMH. The miRNA targetome of coronary artery disease is perturbed by functional polymorphisms identified and prioritized by in-depth bioinformatics analyses exploiting genome-wide association studies. Gene (2016) 594:74–81. 10.1016/j.gene.2016.08.054 27596011

[35] Huan T Rong J Liu C Zhang X Tanriverdi K Joehanes R. Genome-wide identification of microRNA expression quantitative trait loci. Nat Commun. (2015) 6:6601. 10.1038/ncomms7601 25791433 PMC4369777

[36] Civelek M Hagopian R Pan C Che N Yang WP Kayne PS. Genetic regulation of human adipose microrna expression and its consequences for metabolic traits. Hum Mol Genet. (2013) 22:3023–37. 10.1093/hmg/ddt159 23562819 PMC3699064

[37] Ballantyne RL Zhang X Nu-ez S Xue C Zhao W Reed E. Genome-wide interrogation reveals hundreds of long intergenic noncoding RNAs that associate with cardiometabolic traits. Hum Mol Genet. (2016) 25:3125–41. 10.1093/hmg/ddw154 27288454 PMC5181595

[38] McPherson R Pertsemlidis A Kavaslar N Stewart A Roberts R Cox DR. A common allele on chromosome 9 associated with coronary heart disease. Science (2007) 316:1488–91. 10.1126/science.1142447 17478681 PMC2711874

[39] Putku M Kals M Inno R Kasela S Org R KoŽich V. CDH13 promoter SNPs with pleiotropic effect on cardiometabolic parameters represent methylation QTLs. Hum Genet. (2015) 134:291–303. 10.1007/s00439-014-1521-6 25543204 PMC4318987

[40] Chen G Yao C Hwang SJ Liu C Song C Huan T Abstract 18806: integrated proteomic analysis of cardiovascular disease reveals novel protein quantitative trait loci. Circulation (2016).

[41] Hartiala JA Tang WH Wang Z Crow AL Stewart AF Roberts R. Genome-wide association study and targeted metabolomics identifies sex-specific association of CPS1 with coronary artery disease. Nat Commun. (2016) 7:10558. 10.1038/ncomms10558 26822151 PMC4740183

[42] Kraus WE Muoio DM Stevens R Craig D Bain JR Grass E. Metabolomic quantitative trait loci (mQTL) mapping implicates the ubiquitin proteasome system in cardiovascular disease pathogenesis. PLoS Genet. (2015) 11:e1005553. 10.1371/journal.pgen.1005553 26540294 PMC4634848

[43] Laurila PP Surakka I Sarin AP Yetukuri L Hyötyläinen T Söderlund S. Genomic, transcriptomic, and lipidomic profiling highlights the role of inflammation in individuals with low high-density lipoprotein cholesterolsignificance. Arteriosc Thromb Vasc Biol. (2013) 33:847–57. 10.1161/ATVBAHA.112.300733 23413431

[44] Zhu Y Gu X Xu C. A mitochondrial DNA A8701G mutation associated with maternally inherited hypertension and dilated cardiomyopathy in a Chinese pedigree of a consanguineous marriage. Chin Med J. (2016) 129:259–66. 10.4103/0366-6999.174491 26831225 PMC4799567

[45] Kofler B Mueller EE Eder W Stanger O Maier R Weger M. Mitochondrial DNA haplogroup T is associated with coronary artery disease and diabetic retinopathy: a case control study. BMC Med Genet. (2009) 10:35. 10.1186/1471-2350-10-35 19383124 PMC2676278

[46] Mueller EE Eder W Ebner S Schwaiger E Santic D Kreindl T. The mitochondrial T16189C polymorphism is associated with coronary artery disease in Middle European populations. PLoS ONE (2011) 6:e16455. 10.1371/journal.pone.0016455 21298061 PMC3027676

[47] Jia Z Wang X Qin Y Xue L Jiang P Meng Y. Coronary heart disease is associated with a mutation in mitochondrial tRNA. Hum Mol Genet. (2013) 22:4064–73. 10.1093/hmg/ddt256 23736300 PMC3781636

[48] Kumar V Westra HJ Karjalainen J Zhernakova DV Esko T Hrdlickova B. Human disease-associated genetic variation impacts large intergenic non-coding RNA expression. PLoS Genet. (2013) 9:e1003201. 10.1371/journal.pgen.1003201 23341781 PMC3547830

[49] Grubert F Zaugg JB Kasowski M Ursu O Spacek DV Martin AR. Genetic control of chromatin states in humans involves local and distal chromosomal interactions. Cell (2015) 162:1051–65. 10.1016/j.cell.2015.07.048 26300125 PMC4556133

[50] Garnier S Truong V Brocheton J Zeller T Rovital M Wild PS. Genome-wide haplotype analysis of cis expression quantitative trait loci in monocytes. PLoS Genet. (2013) 9:e1003240. 10.1371/journal.pgen.1003240 23382694 PMC3561129

[51] Rotival M Zeller T Wild PS Maouche S Szymczak S Schillert A. Integrating genome-wide genetic variations and monocyte expression data reveals trans-regulated gene modules in humans. PLoS Genet. (2011) 7:e1002367. 10.1371/journal.pgen.1002367 22144904 PMC3228821

[52] Lempiäinen H Brænne I Michoel T Tragante V Vilne B Webb TR. Network analysis of coronary artery disease risk genes elucidates disease mechanisms and druggable targets. Sci Rep. (2018) 8:3434. 10.1038/s41598-018-20721-6 29467471 PMC5821758

[53] Hägg S Skogsberg J Lundström J Noori P Nilsson R Zhong H. Multi-organ expression profiling uncovers a gene module in coronary artery disease involving transendothelial migration of leukocytes and lim domain binding 2: the Stockholm Atherosclerosis Gene Expression (STAGE) Study. PLoS Genet (2009) 5:e1000754. 10.1371/journal.pgen.1000754 19997623 PMC2780352

[54] Talukdar HA Foroughi Asl H Jain RK Ermel R Ruusalepp A Franzén O. Cross-tissue regulatory gene networks in coronary artery disease. Cell Syst. (2016) 2:196–208. 10.1016/j.cels.2016.02.002 27135365 PMC4855300

[55] Small EM Frost RJ Olson EN. MicroRNAs add a new dimension to cardiovascular disease. Circulation (2010) 121:1022–32. 10.1161/CIRCULATIONAHA.109.889048 20194875 PMC2847432

[56] Economou EK Oikonomou E Siasos G Papageorgiou N Tsalamandris S Mourouzis K. The role of microRNAs in coronary artery disease: from pathophysiology to diagnosis and treatment. Atherosclerosis (2015) 241:624–33. 10.1016/j.atherosclerosis.2015.06.037 26117399

[57] Archer K Broskova Z Bayoumi AS Teoh JP Davila A Tang Y. Long non-coding RNAs as master regulators in cardiovascular diseases. Int J Mol Sci. (2015) 16:23651–67. 10.3390/ijms161023651 26445043 PMC4632719

[58] El Azzouzi H Doevendans PA Sluijter JP Long non-coding RNAs in heart failure: an obvious lnc. Ann Trans Med. (2016) 4:182 10.21037/atm.2016.05.06 PMC4876274 27275495

[59] Madrigal-Matute J Rotllan N Aranda JF Fernández-Hernando C. MicroRNAs and atherosclerosis. Curr Atherosc Rep. (2013) 15:322. 10.1007/s11883-013-0322-z 23512606 PMC4193541

[60] Malik R Mushtaque RS Siddiqui UA Younus A Aziz MA Humayun C Association between coronary artery disease and microRNA: literature review and clinical perspective. Cureus (2017) 23:e1188 10.7759/cureus.1188 PMC5441689 28540143

[61] Bulik-Sullivan B Selitsky S Sethupathy P. Prioritization of genetic variants in the microRNA regulome as functional candidates in genome-wide association studies. Hum Mutat. (2013) 34:1049–56. 10.1002/humu.22337 23595788 PMC3807557

[62] Ghaedi H Bastami M Jahani MM Alipoor B Tabasinezhad M Ghaderi O. A bioinformatics approach to the identification of variants associated with Type 1 and Type 2 diabetes mellitus that reside in functionally validated miRNAs binding sites. Biochem Genet. (2016) 54:211–21. 10.1007/s10528-016-9713-5 26820452

[63] Cloonan N Wani S Xu Q Gu J Lea K Heater S. MicroRNAs and their isomiRs function cooperatively to target common biological pathways. Genome Biol. (2011) 12:R126. 10.1186/gb-2011-12-12-r126 22208850 PMC3334621

[64] Li SC Liao YL Ho MR Tsai KW Lai CH Lin WC Mi-RNA arm selection and isomiR distribution in gastric cancer. BMC Genomics (2012) 13:S13 10.1186/1471-2164-13-S1-S13 PMC3303722 22369582

[65] Meijer HA Smith EM Bushell M. Regulation of miRNA strand selection: follow the leader? Biochem Soc Trans. (2014) 42:1135–40. 10.1042/BST20140142 25110015

[66] Samani NJ Erdmann J Hall AS Hengstenberg C Mangino M Mayer B. Genomewide association analysis of coronary artery disease. N Eng J Med. (2007) 357:443–53. 10.1056/NEJMoa072366 17634449 PMC2719290

[67] Schunkert H Götz A Braund P McGinnis R Tregouet DA Mangino M. Repeated replication and a prospective meta-analysis of the association between chromosome 9p21.3 and coronary artery disease. Circulation (2008) 117:1675–84. 10.1161/CIRCULATIONAHA.107.730614 18362232 PMC2689930

[68] Holdt LM Beutner F Scholz M Gielen S Gäbel G Bergert H. ANRIL expression is associated with atherosclerosis risk at chromosome 9p21. Arteriosc Thromb Vasc Biol. (2010) 30:620–7. 10.1161/ATVBAHA.109.196832 20056914

[69] Holdt LM Stahringer A Sass K Pichler G Kulak NA Wilfert W. Circular non-coding RNA ANRIL modulates ribosomal RNA maturation and atherosclerosis in humans. Nat Commun. (2016) 7:12429. 10.1038/ncomms12429 27539542 PMC4992165

[70] Langley SR Dwyer J Drozdov I Yin X Mayr M. Proteomics: from single molecules to biological pathways. Cardiovasc Res. (2012) 97:612–22. 10.1093/cvr/cvs346 23180722 PMC3583257

[71] Dupont A Pinet F. The proteome and secretome of human arterial smooth muscle cell. Cardiovasc Proteom. (2007) 357:225–33. 10.1385/1-59745-214-9:225 17172691

[72] Burkhart JM Vaudel M Gambaryan S Radau S Walter U Martens L. The first comprehensive and quantitative analysis of human platelet protein composition allows the comparative analysis of structural and functional pathways. Blood (2012) 120:73–82. 10.1182/blood-2012-04-416594 22869793

[73] Neisius U Koeck T Mischak H Rossi SH Olson E Carty DM. Urine proteomics in the diagnosis of stable angina. BMC Cardiovasc Disord. (2016) 16:70. 10.1186/s12872-016-0246-y 27095611 PMC4837614

[74] Evers BM Rodriguez-Navas C Tesla RJ Prange-Kiel J Wasser CR Yoo KS. Lipidomic and transcriptomic basis of lysosomal dysfunction in progranulin deficiency. Cell Rep. (2017) 20:2565–74. 10.1016/j.celrep.2017.08.056 28903038 PMC5757843

[75] Krumsiek J Bartel J Theis FJ. Computational approaches for systems metabolomics. Curr Opin Biotechnol. (2016) 39:198–206. 10.1016/j.copbio.2016.04.009 27135552

[76] Teupser D Baber R Ceglarek U Scholz M Illig T Gieger C Genetic regulation of serum phytosterol levels and risk of coronary artery diseaseclinical perspective. Circulation (2010) 3:331–9. 10.1161/CIRCGENETICS.109.907873 20529992

[77] Shah SH Sun JL Stevens RD Bain JR Muehlbauer MJ Pieper KS. Baseline metabolomic profiles predict cardiovascular events in patients at risk for coronary artery disease. Am Heart J. (2012) 163:844–50. 10.1016/j.ahj.2012.02.005 22607863

[78] Krishnan S Huang J Lee H Guerrero A Berglund L Anuurad E. Combined high-density lipoprotein proteomic and glycomic profiles in patients at risk for coronary artery disease. J Proteome Res. (2015) 14:5109–18. 10.1021/acs.jproteome.5b00730 26535788

[79] Feng Q Liu Z Zhong S Li R Xia H Jie Z. Integrated metabolomics and metagenomics analysis of plasma and urine identified microbial metabolites associated with coronary heart disease. Sci Rep. (2016) 6:22525. 10.1038/srep22525 26932197 PMC4773756

[80] Trainor PJ Hill BG Carlisle SM Rouchka EC Rai SN Bhatnagar A. Systems characterization of differential plasma metabolome perturbations following thrombotic and non-thrombotic myocardial infarction. J Proteom. (2017) 160:38–46. 10.1016/j.jprot.2017.03.014 28341595 PMC5496773

[81] Org E Blum Y Kasela S Mehrabian M Kuusisto J Kangas AJ. Relationships between gut microbiota, plasma metabolites, and metabolic syndrome traits in the METSIM cohort. Genome Biol. (2017) 18:70. 10.1186/s13059-017-1194-2 28407784 PMC5390365

[82] Ranjan R Rani A Metwally A McGee HS Perkins DL. Analysis of the microbiome: advantages of whole genome shotgun versus 16S amplicon sequencing. Biochem Biophys Res Commun. (2016) 469:967–77. 10.1016/j.bbrc.2015.12.083 26718401 PMC4830092

[83] Caesar R Fåk F Bäckhed F. Effects of gut microbiota on obesity and atherosclerosis via modulation of inflammation and lipid metabolism. J Int Med. (2010) 268:320–8. 10.1111/j.1365-2796.2010.02270.x 21050286

[84] Le Chatelier E Nielsen T Qin J Prifti E Hildebrand F Falony G Richness of human gut microbiome correlates with metabolic markers. Nature (2013) 500:541–6. 10.1038/nature12506 23985870

[85] Emoto T Yamashita T Kobayashi T Sasaki N Hirota Y Hayashi T. Characterization of gut microbiota profiles in coronary artery disease patients using data mining analysis of terminal restriction fragment length polymorphism: gut microbiota could be a diagnostic marker of coronary artery disease. Heart Vessels (2016) 32:39–46. 10.1007/s00380-016-0841-y 27125213

[86] Senthong V Li XS Hudec T Coughlin J Wu Y Levison B. Plasma trimethylamine N-Oxide, a gut microbe–generated phosphatidylcholine metabolite, is associated with atherosclerotic burden. J Am Coll Cardiol. (2016) 67:2620–8. 10.1016/j.jacc.2016.03.546 27256833 PMC4893167

[87] Fu J Bonder MJ Cenit MC Tigchelaar EF Maatman A Dekens JA. The gut microbiome contributes to a substantial proportion of the variation in blood lipids. Circ Res. (2015) 117:817–24. 10.1161/CIRCRESAHA.115.306807 26358192 PMC4596485

[88] Blekhman R Goodrich JK Huang K Sun Q Bukowski R Bell JT. Host genetic variation impacts microbiome composition across human body sites. Genome Biol. (2015) 16:191. 10.1186/s13059-015-0759-1 26374288 PMC4570153

[89] Zhu J Sova P Xu Q Dombek KM Xu EY Vu H. Stitching together multiple data dimensions reveals interacting metabolomic and transcriptomic networks that modulate cell regulation. PLoS Biol. (2012) 10:e1001301. 10.1371/journal.pbio.1001301 22509135 PMC3317911

[90] Miller CL Pjanic M Wang T Nguyen T Cohain A Lee JD. Integrative functional genomics identifies regulatory mechanisms at coronary artery disease loci. Nat Commun. (2016) 7:12092. 10.1038/ncomms12092 27386823 PMC4941121

[91] Ballinger SW. Mitochondrial dysfunction in cardiovascular disease. Free Radic Biol Med. (2005) 38:1278–95. 10.1016/j.freeradbiomed.2005.02.014 15855047

[92] Kazuno AA Munakata K Nagai T Shimozono S Tanaka M Yoneda M. Identification of mitochondrial DNA polymorphisms that alter mitochondrial matrix pH and intracellular calcium dynamics. PLoS Genet. (2006) 2:e128. 10.1371/journal.pgen.0020128 16895436 PMC1534079

[93] Baccarelli AA Byun HM. Platelet mitochondrial dna methylation: a potential new marker of cardiovascular disease. Clin Epigenet. (2015) 7:44. 10.1186/s13148-015-0078-0 25901189 PMC4404685

[94] Skogsberg J Lundström J Kovacs A Nilsson R Noori P Maleki S. Transcriptional profiling uncovers a network of cholesterol-responsive atherosclerosis target genes. PLoS Genet (2008) 4:e1000036. 10.1371/journal.pgen.1000036 18369455 PMC2265530

[95] Björkegren JL Hägg S Talukdar HA Foroughi Asl H Jain RK Cedergren C. Plasma cholesterol-induced lesion networks activated before regression of early, mature, and advanced atherosclerosis. PLoS Genet (2014) 10:e1004201. 10.1371/journal.pgen.1004201 24586211 PMC3937269

[96] Vilne B Skogsberg J Foroughi Asl H Talukdar HA Kessler T Björkegren JLM. Network analysis reveals a causal role of mitochondrial gene activity in atherosclerotic lesion formation. Atherosclerosis (2017) 267:39–48. 10.1016/j.atherosclerosis.2017.10.019 29100060

[97] Franco-Obregón A Gilbert JA. The microbiome-mitochondrion connection: common ancestries, common mechanisms, common goals. mSystems (2017) 2:e00018–17. 10.1128/mSystems.00018-17 28497122 PMC5425687

[98] Smith DR. The past, present and future of mitochondrial genomics: have we sequenced enough mtDNAs? Brief Func Genom. (2016) 15:47–54. 10.1093/bfgp/elv027 26117139 PMC4812591

[99] Stefely JA Kwiecien NW Freiberger EC Richards AL Jochem A Rush MJP. Mitochondrial protein functions elucidated by multi-omic mass spectrometry profiling. Nat Biotechnol. (2016) 34:1191–7. 10.1038/nbt.3683 27669165 PMC5101133

[100] Picard M McEwen BS Epel ES Sandi C. An energetic view of stress: focus on mitochondria. Front Neuroendocrinol. (2018) 49:72–85. 10.1016/j.yfrne.2018.01.001 29339091 PMC5964020

[101] Huang S Chaudhary K Garmire LX. More is better: recent progress in multi-omics data integration methods. Front Genet. (2017) 8:84. 10.3389/fgene.2017.00084 28670325 PMC5472696

